# Chitosan–PEG Nanoparticles for Co-Delivery of Paclitaxel and KRAS G12D-Directed siRNA to Pancreatic Cancer Cells

**DOI:** 10.3390/ijms27167285

**Published:** 2026-08-15

**Authors:** Yu-Ting Chien, Jianxi Huang, Yuanhao Zhao, Yumeng Zhou, Miqin Zhang, Qingxin Mu

**Affiliations:** 1 Department of Pharmaceutics, University of Washington, Seattle, WA 98195, USA; kingwang0022@gmail.com; 2 Department of Materials Science and Engineering, University of Washington, Seattle, WA 98195, USA; jianxih@uw.edu (J.H.); yuanhaoz@uw.edu (Y.Z.); zhouyum6@uw.edu (Y.Z.)

**Keywords:** chemotherapy, siRNA, cancer, co-delivery, polymeric nanoparticles, KRAS, G12D

## Abstract

Pancreatic ductal adenocarcinoma (PDAC) remains one of the most lethal malignancies due to limited responsiveness to chemotherapy and the high prevalence of oncogenic Kirsten rat sarcoma viral oncogene homolog (KRAS) mutations. Co-delivery of cytotoxic agents and small interfering RNA (siRNA) is a potential combination strategy, but the two cargos have distinct physicochemical and intracellular-delivery requirements. Here, we developed a chitosan–polyethylene glycol (CP)-based polymeric nanoparticle platform for the cotreatment of paclitaxel (PTX) and small interfering RNA (siRNA) targeting KRAS G12D mutation. PTX was first modified to PTX-COOH through an ester-containing succinate linker and then covalently conjugated to the polymer backbone through amide bond formation, enabling stable nanoparticle formation and subsequent electrostatic complexation with siRNA. The CP-PTX-siRNA nanoparticles demonstrated efficient cellular uptake, while luciferase knockdown by CP-siRNA supported functional siRNA delivery by the CP carrier. In KRAS G12D–mutant pancreatic cancer cells, PTX- and KRAS-targeting siRNA-coloaded nanoparticles resulted in enhanced cytotoxicity compared to single-agent treatments and free drug combinations, with combination index values below 1 indicating calculated synergy under the tested in vitro conditions. Because KRAS knockdown and a non-targeting siRNA control were not assessed in PANC-1 cells, the enhanced cytotoxicity cannot be attributed specifically to KRAS silencing. Across multiple drug-to-siRNA ratios, nanoparticle formulations consistently improved treatment potency. Together, these results support CP-PTX-siRNA nanoparticles as a modular and biocompatible platform for combined PTX/siRNA delivery. This approach provides a versatile strategy for combining chemotherapeutic agents with RNA-based therapeutics in PDAC, supporting further development of these polymeric nanocarriers for combination cancer therapy.

## 1. Introduction

Pancreatic ductal adenocarcinoma (PDAC), a most common form of pancreatic cancer, is one of the most aggressive and treatment-resistant malignancies, with a five-year survival rate remaining in the low double digits despite decades of therapeutic development [1,2,3]. Late-stage diagnosis, rapid disease progression, and poor responsiveness to systemic therapies contribute to its devastating clinical outcomes [4,5]. Although combination chemotherapy regimens have modestly improved survival, durable responses remain uncommon, and most patients ultimately develop progressive disease [6,7,8]. These challenges highlight the need for therapeutic strategies that move beyond conventional cytotoxic approaches and directly target the molecular drivers of PDAC.

Chemotherapy remains a cornerstone of PDAC treatment for patients with unresectable or metastatic diseases [7,8]. Paclitaxel-containing regimens, particularly in albumin-bound formulations, have demonstrated improved response rates and survival compared to gemcitabine monotherapy [9]. However, the therapeutic benefit of paclitaxel-based therapies is frequently limited by dose-limiting toxicity, suboptimal tumor accumulation, and the emergence of drug resistance [10,11]. At the cellular level, PDAC tumors exhibit multiple mechanisms that reduce chemotherapy efficacy, including altered drug transport, enhanced survival signaling, and rapid adaptation to cytotoxic stress [12]. As a result, chemotherapy alone is insufficient to produce sustained disease control in most patients.

A defining molecular feature of PDAC is the near-universal presence of activating mutations in the Kirsten rat sarcoma viral oncogene homolog (KRAS) oncogene, detected in over 90% of cases [13,14]. Among these, the G12D mutation is the most prevalent and plays a central role in driving tumor initiation, maintenance, and therapeutic resistance [15]. Mutant KRAS constitutively activates downstream signaling pathways, including mitogen-activated protein kinase (MAPK) and phosphoinositide 3-kinase–AKT (PI3K–AKT) pathways, promoting uncontrolled proliferation, survival, and resistance to apoptosis [16]. Despite its importance, KRAS has historically been considered a difficult therapeutic target due to its high affinity for guanosine triphosphate (GTP) and guanosine diphosphate (GDP) and the lack of accessible binding pockets for small-molecule inhibitors [17]. Recent advances have yielded promising KRAS-targeted inhibitors, producing significantly prolonged survival in metastatic PDAC patients compared to chemotherapy [18].

Despite encouraging progress in KRAS-targeted small molecule inhibitors, RNA interference remains an alternative and may provide a complementary approach to targeting oncogenic KRAS by suppressing gene expression at the mRNA level [19,20]. Small interfering RNA (siRNA) enables sequence-specific degradation of target transcripts, allowing selective inhibition of mutant KRAS signaling [21]. Preclinical and early clinical studies have demonstrated that KRAS-targeting siRNA can reduce tumor growth and sensitize cancer cells to chemotherapy [22,23]. However, the clinical translation of siRNA-based therapies has been hindered by fundamental delivery challenges. Naked siRNA is rapidly degraded in biological fluids, exhibits poor cellular uptake, and is prone to endosomal entrapment following internalization [21]. These limitations are further compounded when siRNA is combined with chemotherapeutic agents, as the two cargos possess distinct physicochemical properties and pharmacokinetic requirements [24].

Nanoparticle-based delivery systems provide a promising strategy to overcome these barriers by protecting therapeutic cargos and enabling coordinated intracellular delivery [25,26,27,28]. Polymeric nanoparticles, in particular, offer structural stability, tunable surface chemistry, and flexibility in cargo loading. Polymeric systems can also be chemically modified to incorporate multiple functionalities, including drug conjugation, nucleic acid complexation, and surface stabilization [29,30]. These properties make polymeric nanoparticles well suited for combination therapies that integrate small-molecule drugs with nucleic-acid-based agents.

Chitosan is an especially attractive polymeric material for such applications due to its biocompatibility, biodegradability, and high density of primary amine groups [25]. Its cationic nature facilitates electrostatic complexation with negatively charged nucleic acids, enabling efficient siRNA loading and protection [23]. In parallel, chitosan can be chemically modified to accommodate hydrophobic chemotherapeutics through covalent conjugation, allowing stable incorporation of drugs such as paclitaxel [31]. Compared to physical encapsulation approaches, covalent attachment of chemotherapeutics to polymer backbones can improve drug retention and reduce premature release compared with physical encapsulation, although release behavior depends on the specific linker chemistry and formulation context.

Integrating chemotherapy and gene silencing within a single delivery platform offers the potential for synergistic therapeutic effects [32]. Chemotherapy can induce cellular stress and sensitize cancer cells to gene knockdown, while suppression of oncogenic signaling pathways can reduce resistance to cytotoxic agents [33]. However, achieving this synergy remains challenging because small-molecule drugs and nucleic acids possess fundamentally different physicochemical properties and delivery requirements. Hydrophobic chemotherapeutics require stable incorporation to prevent premature release, whereas siRNA is highly hydrophilic, negatively charged, and prone to degradation and endosomal trapping [34]. Consequently, many co-delivery systems suffer from instability or asynchronous intracellular release, limiting therapeutic synergy [35].

In this study, we developed a chitosan–polyethylene-glycol-based polymeric nanoparticle platform for co-delivery of paclitaxel and siRNA. Paclitaxel was first modified to PTX-COOH through an ester-containing succinate linker and then covalently conjugated to the PEG-modified chitosan (CP) backbone through amide-bond formation. The resulting CP-PTX retained cationic functionality for electrostatic siRNA complexation, enabling a two-cargo formulation with tunable PTX:siRNA ratios. We characterized nanoparticle size, surface charge, morphology, and short-term stability; examined CP-PTX-siRNA uptake and intracellular distribution in Pan02 cells; assessed luciferase-siRNA activity using CP-based formulations in Pan02-Luc cells; and evaluated the cytotoxicity of PTX/KRAS G12D-directed siRNA formulations in PANC-1 cells. Because KRAS knockdown and a non-targeting siRNA control were not included in the PANC-1 experiments, the therapeutic studies evaluate formulation-associated cytotoxicity rather than establish sequence-specific KRAS silencing. The present work therefore focuses on the feasibility of the CP platform for co-delivery of PTX/siRNA and on identifying formulation parameters for subsequent mechanistic and in vivo validation.

## 2. Results and Discussion

### 2.1. Design of the CP-PTX Nanoparticles (NPs) Platform

**Figure 1:**
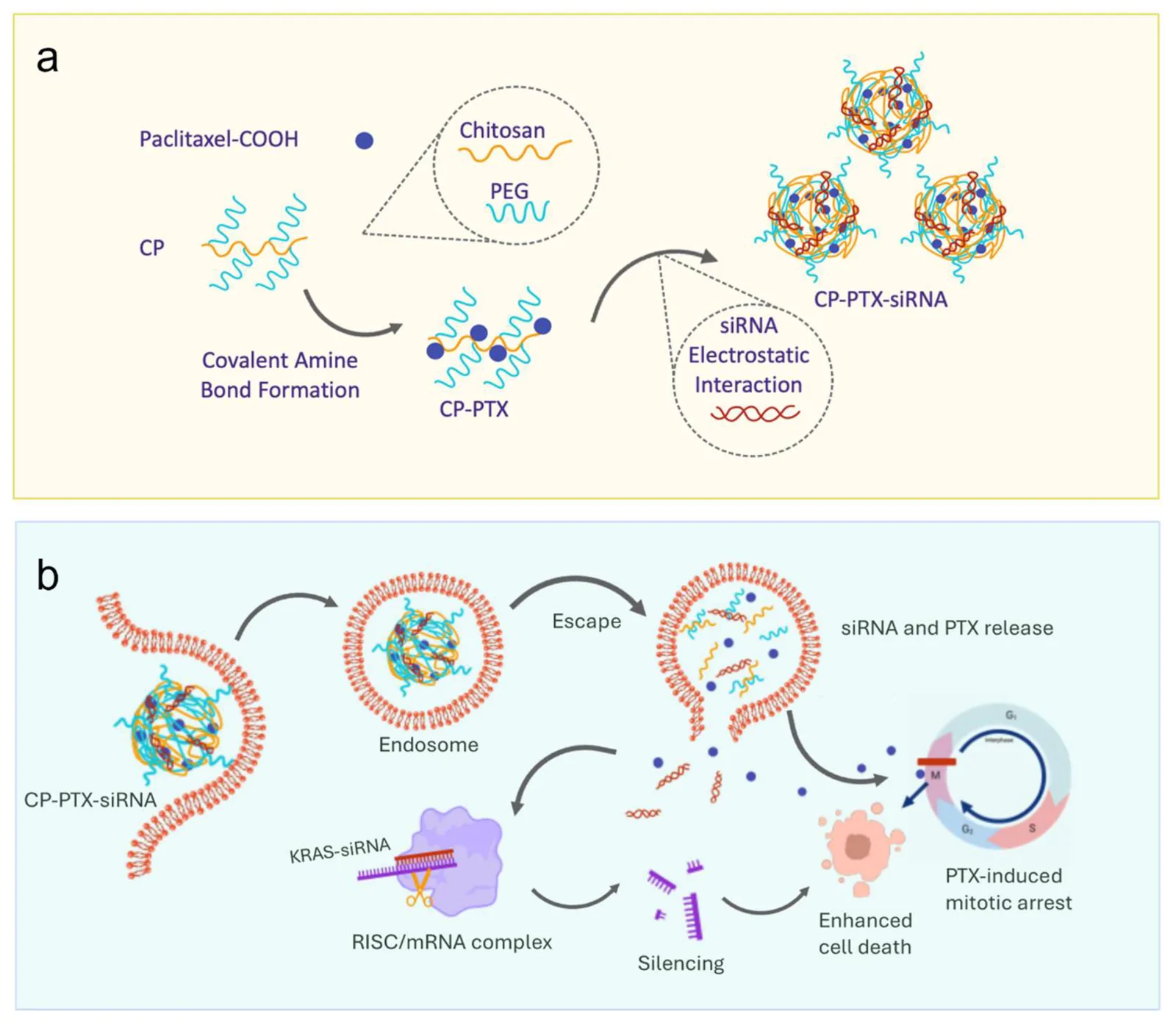
Design and proposed therapeutic mechanism of CP-PTX-siRNA nanoparticles for co-delivery of PTX and siRNA to KRAS-mutant PDAC cells. (**a**) Schematic illustration of NP synthesis. PTX was first modified to PTX-COOH and then covalently conjugated to the CP polymer, composed of chitosan and polyethylene glycol (PEG), to form CP-PTX. The resulting CP-PTX was then complexed with siRNA via electrostatic interaction to generate CP-PTX-siRNA NPs. (**b**) Conceptual intracellular mechanism. Following cellular uptake, the NPs may enter endosomal compartments and subsequently release siRNA into the cytoplasm after intracellular trafficking. The released siRNA may engage the RISC complex and mediate target mRNA silencing. PTX, which is covalently conjugated to the CP backbone, may contribute to cytotoxic stress after intracellular delivery. The combined effects of PTX-mediated cytotoxicity and KRAS-targeting siRNA delivery may enhance cancer cell killing.

We designed a polymeric nanoparticle platform based on chitosan–polyethylene glycol (CP) for the co-delivery of paclitaxel (PTX) and small interfering RNA (siRNA), with the goal of integrating chemotherapy and gene silencing within a single carrier system. The platform architecture was guided by two primary considerations: first, the need for stable incorporation of hydrophobic PTX without premature release, and second, the ability to efficiently complex and deliver siRNA for intracellular gene knockdown. To address these requirements, PTX was covalently conjugated to the polymer backbone, while siRNA was incorporated through electrostatic complexation, enabling coordinated yet modular cargo loading (Figure 1a).

Paclitaxel was first modified with succinic anhydride to generate PTX-COOH through an ester-containing succinate linker and was subsequently conjugated to CP through EDC/NHS-mediated amide bond formation, yielding a CP-PTX construct with retained cationic character. This covalent conjugation strategy distinguishes the CP-PTX platform from nanoparticle systems that rely on physical encapsulation of PTX, which often suffer from burst release and limited drug retention. By anchoring PTX to the polymer backbone, the platform is intended to improve formulation stability, while the PTX release behavior is inferred from previous studies using the same CP-PTX chemistry rather than directly measured in the present CP-PTX-siRNA formulation [36].

The presence of primary amines on the chitosan backbone allows CP-PTX to electrostatically associate with negatively charged siRNA, driving spontaneous nanoparticle assembly in aqueous environments. This two-component design decouples drug loading from nucleic acid complexation, permitting independent optimization of PTX content and siRNA dose without altering the core nanoparticle structure. Such modularity is particularly advantageous for combination therapies, where precise control over drug-to-siRNA ratios is critical for achieving therapeutic synergy.

The optimized platform was applied to deliver KRAS G12D-targeting siRNA, enabling selective post-transcriptional silencing of oncogenic KRAS in pancreatic cancer models. In contrast to several published KRAS-targeting NPs platforms, which utilize peptide-based or peptide-nucleic acid conjugates with limited adaptability or involve complex formulations prone to instability, our CP-based system provides a simplified yet tunable scaffold for multiplexed therapeutic delivery.

Figure 1b illustrates the proposed mechanism of action of the designed NPs in KRAS-mutant cancer cells. Briefly, following cellular uptake, the nanoparticles are expected to traffic through endosomal and lysosomal compartments. Based on the ester-containing succinate linker and prior studies using the same CP-PTX chemistry [36], acidic intracellular conditions may promote linker cleavage and PTX release. Once released intracellularly, PTX may exert its cytotoxic effect by stabilizing microtubules and inhibiting mitotic spindle disassembly, resulting in mitotic arrest and apoptosis of rapidly dividing tumor cells.

Simultaneously, the KRAS G12D-targeting siRNA may dissociate from the NP matrix and become incorporated into the RNA-induced silencing complex (RISC) in the cytoplasm. Guided by sequence complementarity, the siRNA-RISC complex may bind and degrade KRAS G12D mutant mRNA, leading to suppression of downstream oncogenic signaling pathways such as MAPK and PI3K/AKT. This post-transcriptional silencing may inhibit tumor cell proliferation and survival and reduce intrinsic resistance mechanisms to chemotherapeutics [37,38].

Importantly, these two agents are proposed to function through complementary mechanisms: PTX may disrupt the mitotic process, while KRAS G12D-targeting siRNA may suppress oncogenic signaling pathways associated with tumor cell proliferation and survival. By combining these therapeutic modalities within a single nanoparticle platform, CP-PTX-siRNA NPs may enhance cytotoxic activity compared with single-agent or admixed treatments. Overall, our CP-PTX-siRNA platform integrates chemotherapeutic drug conjugation and targeted siRNA complexation in a single, biocompatible construct, distinguishing it from conventional systems and highlighting its potential for further development in combination nanomedicine.

### 2.2. Synthesis and Structural Validation of CP-PTX

**Figure 2:**
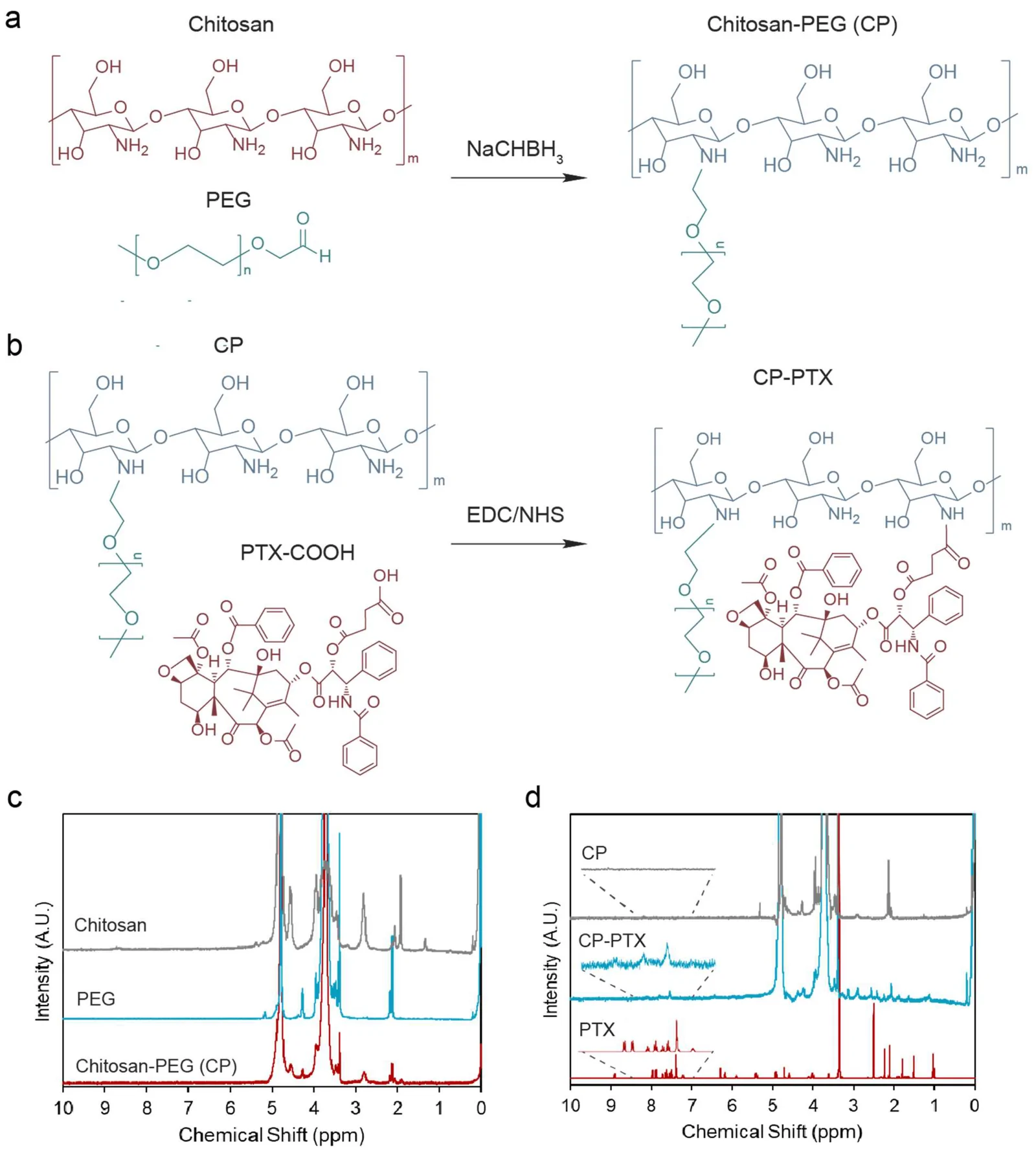
Synthesis and characterization of CP and CP-PTX. Schematic illustration of the chemical conjugation of (**a**) chitosan onto PEG in the presence of sodium cyanoborohydride and (**b**) PTX-COOH to the CP polymer through EDC/NHS-mediated amide bond formation. PTX-COOH was generated by succinic anhydride modification of PTX through an ester-containing succinate linker. (**c**,**d**) Representative ^1^H NMR spectra of chitosan, PEG, CP, CP-PTX, and free PTX confirming successful conjugation.

The CP-PTX conjugate was synthesized to generate a polymeric backbone capable of stably incorporating paclitaxel (PTX) while retaining cationic functionality for siRNA complexation. The synthesis proceeded in two stages: formation of the chitosan–polyethylene glycol (CP) carrier (Figure 2a), followed by PTX modification and covalent conjugation to CP (Figure 2b). The formation of CP followed a previously established chitosan–PEG conjugation protocol [39]. Chitosan was grafted onto PEG through amine-reactive coupling chemistry, yielding a CP polymer that combines the biocompatibility and steric stabilization of PEG with the cationic, nucleic-acid-binding properties of chitosan. Successful formation of CP was confirmed by ^1^H nuclear magnetic resonance (^1^H NMR) spectroscopy, which showed characteristic proton signals corresponding to both PEG (4–4.5 ppm) and chitosan (2.5–3 ppm), indicating effective conjugation (Figure 2c). The glucosamine ring protons of chitosan appeared mainly in the 3–4 ppm region, which partially overlaps with PEG-related methylene proton signals, and therefore this region was interpreted as supporting the presence of both polymer components rather than as an isolated diagnostic peak for conjugation.

To enable covalent attachment of PTX to CP, paclitaxel was first converted to a carboxylated derivative (PTX-COOH) through succinic anhydride modification. This reaction introduces a carboxyl group through an ester-containing succinate linker while preserving the taxane structure. PTX-COOH was then conjugated to CP through EDC/NHS-mediated amide bond formation between the activated carboxyl group of PTX-COOH and primary amines on the chitosan backbone. This covalent conjugation strategy was selected to improve drug incorporation and potentially reduce premature PTX loss compared with physically encapsulated formulations.

Successful formation of the CP-PTX conjugate was verified through nuclear magnetic resonance (NMR) spectroscopy (Figure 2d). Characteristic signals corresponding to chitosan and PEG appeared at 3.5–4 ppm, while aromatic protons from the PTX moiety were evident between 7–8.2 ppm and were absent in the CP control, supporting successful PTX conjugation. Based on peak integration using TSP as an internal standard, the PTX conjugation level in the purified CP-PTX conjugate was estimated to be approximately 3.21% *w*/*w*. This experimentally determined value was used to calculate the nominal PTX dose and to convert CP-PTX:siRNA mass ratios to PTX:siRNA molar ratios in the subsequently prepared formulations. PTX content was not independently quantified by HPLC in the final CP-PTX-siRNA formulation. Because PTX was covalently attached to CP before siRNA complexation, its conjugation level was not expected to change during nanoparticle assembly.

### 2.3. Physiochemical Properties of CP-PTX-siRNA NPs

**Figure 3:**
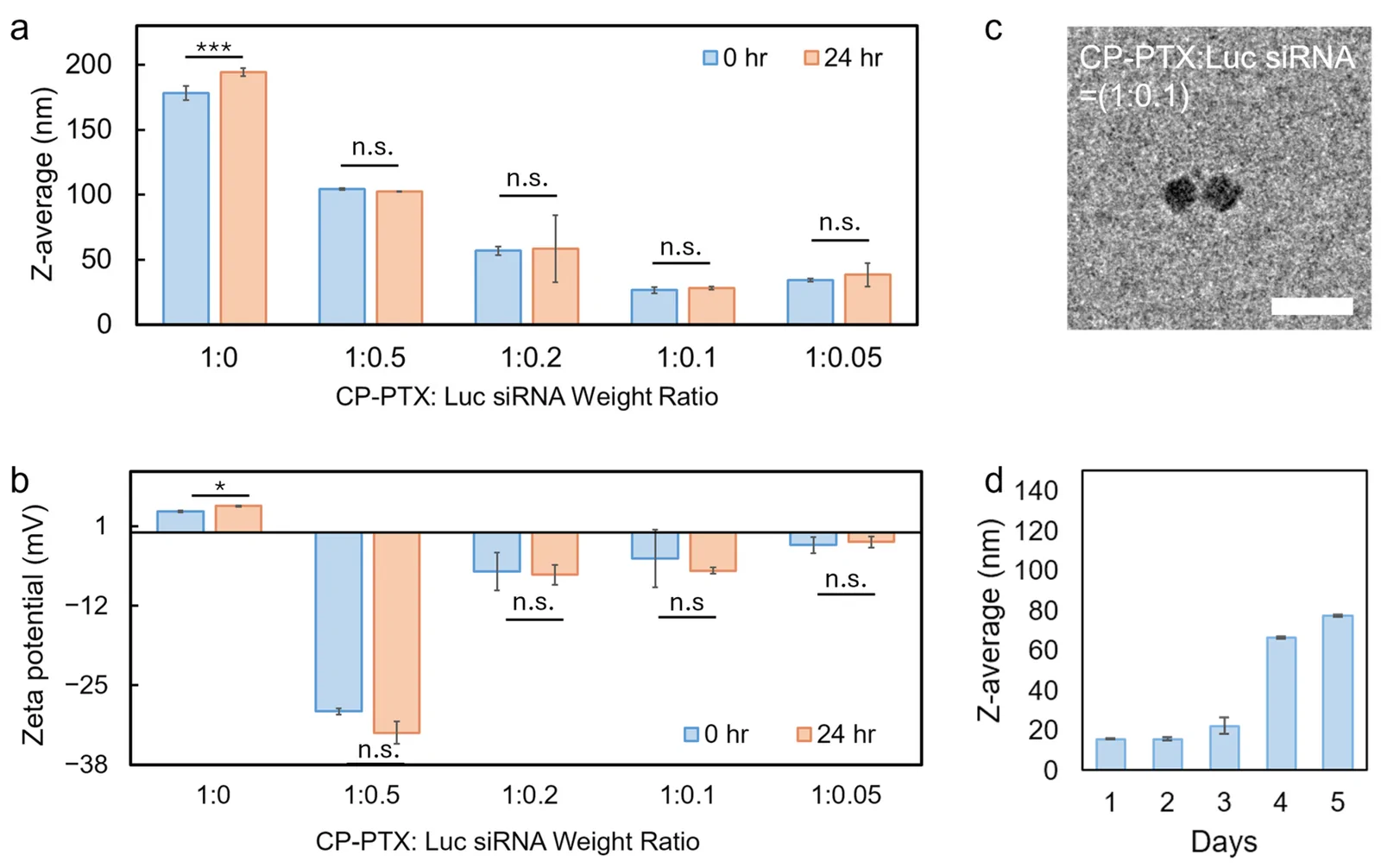
Characterization of CP-PTX-siRNA NPs with various CP-PTX:siRNA weight ratios. Samples were measured immediately after formulation and after 24 h in HEPES buffer, pH 6.8. The data illustrate how the CP-PTX-to-Luc siRNA weight ratio influences hydrodynamic size (**a**), surface charge (**b**), and colloidal stability over time. * *p* < 0.05, *** *p* < 0.001; n.s., not significant. (**c**) TEM image of the selected CP-PTX-siRNA nanoparticles at a CP-PTX:siRNA weight ratio of 1:0.1, showing compact nanoparticle morphology with an average diameter of approximately 21.0 nm. Scale bar represents 50 nm. (**d**) Five-day apparent hydrodynamic size stability of CP-PTX-siRNA nanoparticles in RPMI cell culture medium supplemented with 10% FBS at 37 °C.

After confirming formation of the CP-PTX conjugate, we next evaluated the physicochemical properties of the CP-PTX-siRNA formulations to identify compositions suitable for downstream functional studies (Figure 3). Luciferase-targeting siRNA, which carries a net negative charge, was mixed with cationic CP-PTX at defined mass ratios to promote electrostatic association and nanoparticle assembly. Because the nanoparticles were prepared immediately before use and were not subsequently isolated or purified as a separate final product, siRNA complexation efficiency was not directly quantified. Separation of unbound siRNA from the nanoparticles could disturb the reversible electrostatic equilibrium and alter the formulation being analyzed. Accordingly, changes in hydrodynamic size and zeta potential following siRNA addition were used as physicochemical evidence supporting siRNA association and nanoparticle formation rather than as a quantitative measure of complexation efficiency.

At a 1:0 ratio (no siRNA), particles exhibited a hydrodynamic diameter of 178.18 nm with a slightly positive zeta potential (+3.44 mV). Upon incorporation of siRNA, both particle size and surface charge decreased, indicating effective electrostatic complexation between CP-PTX and siRNA. At a 1:0.2 ratio, the particle size decreased to 56.83 nm and the zeta potential shifted to −6.43 mV. Among all tested formulations, the 1:0.1 mass ratio (PTX:siRNA molar ratio of ~1:0.2) yielded the smallest nanoparticles, with a hydrodynamic diameter of 26.47 nm and a moderately negative zeta potential of −9.76 mV.

To assess nanoparticle stability over time, particle size and zeta potential were measured immediately after formulation (0 h) and after 24 h of incubation in HEPES buffer (Figure 3). For all siRNA-containing formulations (1:0.05–1:0.5), no statistically significant changes in particle size were observed over 24 h (*p* > 0.05), indicating short-term colloidal stability. The measured polydispersity indices ranged from 0.36 to 0.57, indicating relatively broad and heterogeneous particle-size distributions. Although all ratios generated nanoscale nanoparticle profiles, the composition with 1:0.1 CP-PTX:siRNA mass ratio was selected for cellular siRNA delivery studies due to its compact size, favorable surface charge, and colloidal stability, which are desirable for efficient cellular uptake and downstream biological evaluation.

To further characterize the optimized CP-PTX-siRNA formulation, nanoparticle morphology was examined by transmission electron microscopy (TEM). As shown in Figure 3c, the optimized 1:0.1 CP-PTX:siRNA formulation formed discrete, compact nanoparticles with an average diameter of approximately 21.0 nm. This TEM-measured size was consistent with the hydrodynamic diameter measured by DLS in HEPES buffer, supporting the successful formation of nanoscale CP-PTX-siRNA complexes. The slightly smaller size observed by TEM compared with DLS is expected because TEM measures dried nanoparticles, whereas DLS measures the hydrated particle diameter in solution.

We next evaluated the stability of the optimized CP-PTX-siRNA nanoparticles in RPMI cell culture medium supplemented with 10% FBS at 37 °C, over 5 days. The apparent hydrodynamic diameters were 15.6, 15.7, 22.3, 66.4, and 77.4 nm from day 1 to day 5, respectively (Figure 3d). The nanoparticles remained within the nanoscale range throughout the observation period, suggesting that the formulation did not undergo severe macroscopic aggregation in serum-containing medium. The smaller apparent size during the first three days compared with the size measured in HEPES buffer may reflect ionic-strength-mediated charge screening, changes in the hydration layer, or contributions from serum/media components during DLS measurement. The gradual increase in size observed on days 4 and 5 may indicate serum protein adsorption, partial nanoparticle rearrangement, or limited aggregation after prolonged incubation at 37 °C. Together, these results suggest that CP-PTX-siRNA nanoparticles maintain short-term colloidal stability in biologically relevant medium, while prolonged incubation in serum-containing conditions may alter their apparent hydrodynamic behavior.

### 2.4. Cellular Uptake and siRNA-Delivery Studies of CP-PTX-siRNA NPs

To evaluate the intracellular delivery capability of CP-PTX-siRNA nanoparticles (NPs), we first assessed cellular uptake in murine pancreatic cancer cells (Pan02). Pan02 cells were selected as a relevant preclinical model of pancreatic cancer, as they are commonly used in syngeneic tumor studies and enable direct translation of in vitro findings to subsequent in vivo experiments. For functional studies, a luciferase-expressing variant (Pan02-Luc) was employed to allow quantitative assessment of siRNA-mediated gene silencing through bioluminescence readouts.

**Figure 4:**
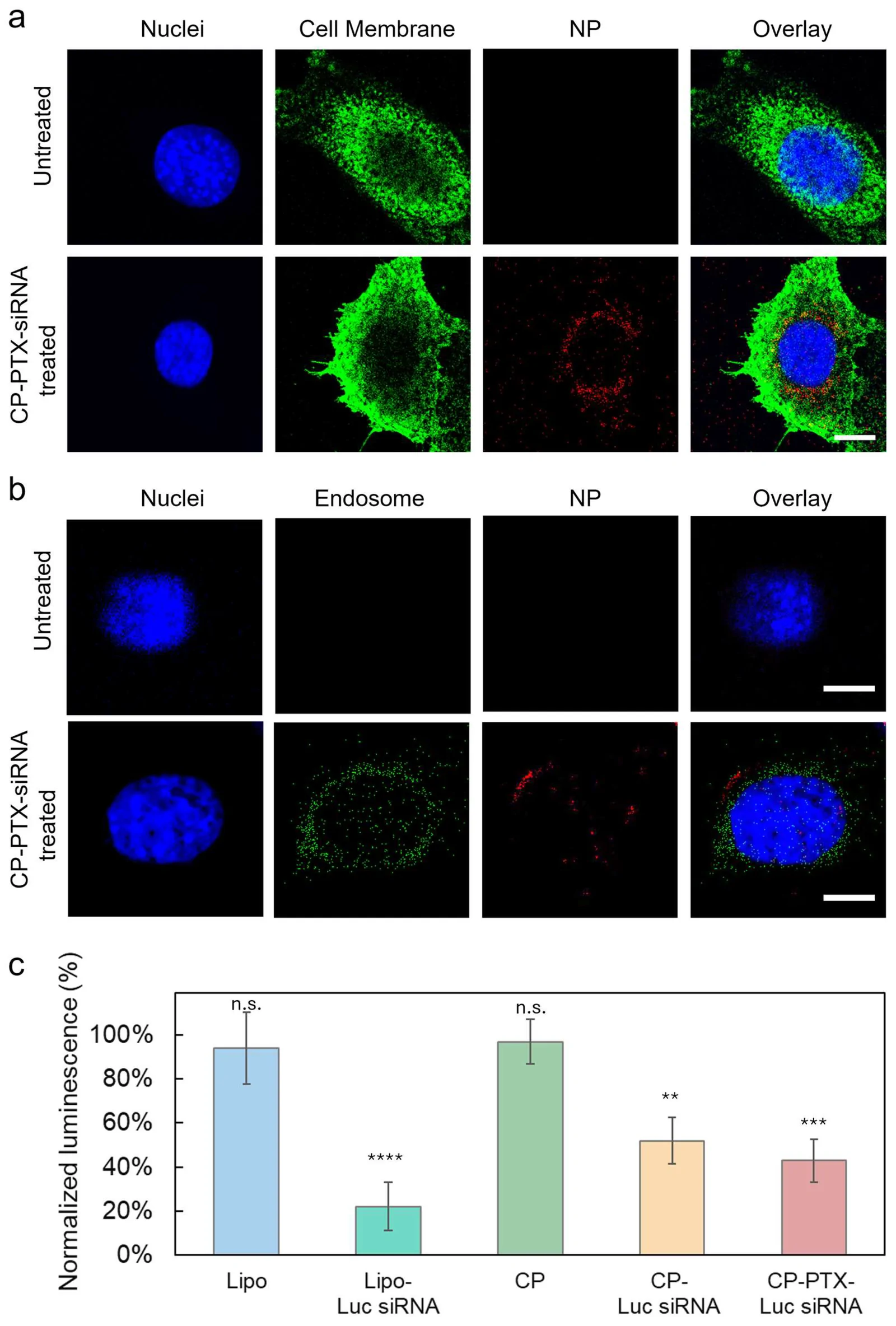
Cellular uptake, intracellular localization, and siRNA-delivery studies of CP-PTX-siRNA nanoparticles. (**a**) Confocal laser scanning microscopy images of Pan02 cells following incubation with Cy5-labeled CP-PTX-siRNA nanoparticles (NPs) at a CP-PTX mass ratio of 1:0.1 for 2 h. Untreated cells were used as a control. Nuclei are stained with DAPI (blue), cell membranes with WGA-AF555 (green), and nanoparticles are shown in red (Cy5). The overlay images demonstrate efficient cellular internalization and cytoplasmic localization of CP-PTX-siRNA NPs. Scale bar: 10 μm. (**b**) Single-timepoint intracellular localization of CP-PTX-siRNA NPs in Pan02 cells. Cells were treated with Cy5-labeled CP-PTX-Luc siRNA NPs for 4 h, followed by LysoTracker staining for 1 h before fixation and imaging. Nuclei are stained with DAPI (blue), endosomal/lysosomal compartments are shown in green, and nanoparticles are shown in red. The merged images show limited overlap between LysoTracker-positive compartments and Cy5-labeled NPs, indicating possible partial spatial separation at this single timepoint; this qualitative observation does not establish endosomal/lysosomal escape. Scale bar: 10 μm. (**c**) Functional delivery of luciferase-targeting siRNA evaluated by bioluminescence imaging (BLI) in Pan02-Luc cells. Cells were treated with Lipo, Lipo–Luc siRNA, CP, CP–Luc siRNA, or CP-PTX–Luc siRNA for 24 h. The luciferase siRNA concentration was fixed at 200 nM for all siRNA-containing groups. For control groups, Lipo and CP were applied at the same amounts used in their corresponding siRNA formulations. Luminescence intensity was normalized to untreated controls (100%). Statistical significance was determined by one-way ANOVA followed by Dunnett’s multiple comparisons test against the untreated control (** *p* < 0.01, *** *p* < 0.001, **** *p* < 0.0001; n.s., not significant).

Cellular uptake was examined using confocal laser scanning microscopy following incubation of Pan02 cells with Cy5-labeled CP-PTX-siRNA NPs at the optimized CP-PTX:siRNA mass ratio of 1:0.1. As shown in Figure 4a, strong intracellular fluorescence was observed after treatment, indicating efficient nanoparticle association and internalization. The fluorescent signal was primarily localized within the cytoplasmic region, consistent with endocytic uptake rather than nonspecific surface binding. In contrast, untreated cells exhibited negligible background fluorescence.

To further evaluate whether internalized CP-PTX-siRNA NPs could escape from endosomal/lysosomal compartments, Pan02 cells were treated with Cy5-labeled CP-PTX-Luc siRNA NPs for 4 h and stained with LysoTracker before confocal imaging. As shown in Figure 4b, the merged images showed limited visual overlap between LysoTracker-positive compartments and Cy5-labeled nanoparticles, with some nanoparticle-associated signals appearing spatially separated from endosomal/lysosomal compartments. This qualitative observation at a single 4 h timepoint suggests possible partial separation of internalized CP-PTX-siRNA nanoparticles from endosomal/lysosomal compartments after cellular uptake. As endosomal entrapment is a major barrier for siRNA delivery, this partial endosomal/lysosomal escape may contribute to the functional siRNA knockdown [40]. The extent and kinetics of endosomal escape will be analyzed from future mechanistic studies, including quantitative colocalization analysis.

To determine whether internalized nanoparticles could mediate functional siRNA delivery, we next evaluated luciferase knockdown in Pan02-Luc cells using bioluminescence imaging (BLI). Luciferase expression was quantified 24 h after treatment and normalized to untreated controls. As shown in Figure 4c, CP alone had minimal effect on luciferase expression (97% ± 10.0%), confirming its biocompatibility. Lipofectamine-mediated siRNA delivery (Lipo-siRNA) served as a positive control and reduced luciferase expression to 22% ± 11.0%. Importantly, CP-siRNA achieved 52% ± 10.4% luciferase expression, demonstrating that the CP platform can effectively deliver functional siRNA. Notably, CP-PTX-siRNA NPs further reduced luciferase expression to 43% ± 9.8%, indicating that conjugation of paclitaxel to the polymer backbone does not compromise siRNA delivery or gene silencing efficiency. As luminescence was not normalized to a parallel measurement of cell viability, cell number, or total protein, the additional signal reduction observed for CP-PTX-siRNA cannot be attributed solely to greater siRNA-mediated knockdown. PTX-induced cytotoxic or metabolic effects might also have contributed to the reduced luminescence.

Taken together, Figure 4 shows intracellular uptake of CP-PTX-siRNA nanoparticles in Pan02 cells and a functional luciferase-siRNA readout for the CP-siRNA formulation in Pan02-Luc cells. The single 4 h LysoTracker observation shows only possible partial spatial separation and does not establish endosomal escape. In addition, the PTX-containing luciferase group is confounded by potential effects of PTX on cell number or metabolism. These limitations were considered when interpreting the subsequent PANC-1 cytotoxicity studies.

### 2.5. Cytotoxicity of PTX/KRAS G12D-Directed siRNA Formulations in PANC-1 Cells

To evaluate cytotoxicity associated with PTX/KRAS G12D-directed siRNA combinations, PANC-1 cells were treated with PTX, KRAS G12D-directed siRNA delivered by Lipofectamine, admixed PTX plus siRNA, or CP-PTX-KRAS G12D siRNA nanoparticles at varying concentrations. PANC-1 cells carry the KRAS G12D mutation and were used as the human PDAC cytotoxicity model [41]. Uptake and luciferase-siRNA studies, however, were performed only in Pan02/Pan02-Luc cells. The absence of a cell-specific targeting ligand does not establish equivalent uptake or intracellular delivery in PANC-1 cells; treating the Pan02 observations as broadly applicable to PANC-1 is therefore an explicit, untested assumption and a limitation of the present study. Cell viability was measured after 72 h and normalized to untreated controls. The longer incubation period was selected to capture delayed effects of siRNA exposure and PTX treatment on cell viability.

**Figure 5:**
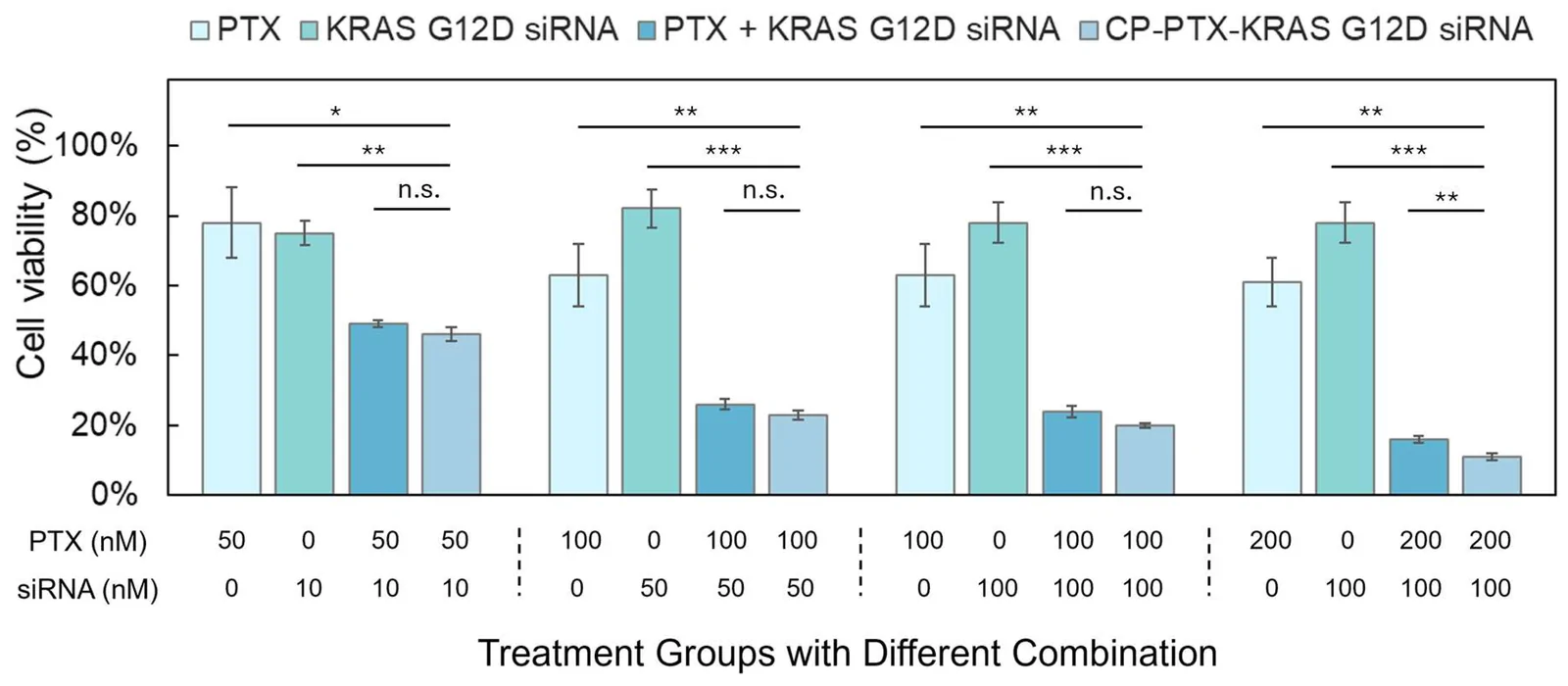
Cell viability across groups with varying concentrations of PTX and KRAS siRNA in PANC-1 Cells after 72 h of incubation. PANC-1 cells were seeded in a 96-well plate (1 × 10^4^ cells/well) and cultured for 24 h prior to treatment. PANC-1 cells were seeded in 96-well plates (1 × 10^4^ cells/well) and cultured for 24 h before treatment with PTX, Lipo-siRNA, PTX + Lipo-siRNA, or CP-PTX-siRNA at the indicated concentrations. Bars represent mean ± SD from three technical replicate wells within a single experiment. For the displayed matched-dose pairwise comparisons, two-sided unpaired t tests were performed and *p* values were adjusted across the displayed comparison family using the Holm–Šídák method. Because only technical replicates from one experiment were available, these pairwise tests are exploratory; the adjusted *p* values describe within-experiment technical variation and do not establish biological reproducibility. Significance levels are * *p* < 0.05, ** *p* < 0.01, *** *p* < 0.001, n.s., not significant.

As shown in Figure 5, single-agent treatments exhibited moderate dose-dependent cytotoxicity. Free PTX at concentrations of 50 nM and 100 nM resulted in cell viabilities of 78% (±13.4%) and 63% (±14.1%), respectively. Increasing PTX concentration to 200 nM slightly decreased viability further, to 61% (±11.3%). Treatment with KRAS-targeting siRNA alone had limited cytotoxicity across the tested concentrations (10 nM: 75% ± 4.7%, 50 nM: 82% ± 6.5%, 100 nM: 78% ± 7.6%).

Combining free PTX and siRNA produced lower cell viability than either tested single agent at several matched dose combinations. For example, 50 nM PTX + 10 nM siRNA yielded 49% ± 1.9% viability, and 100 nM PTX + 50 nM siRNA yielded 26% ± 5.4% viability. Viability declined further at higher combination doses (Figure 5). These selected-dose comparisons show greater cytotoxicity of the combinations than the corresponding single-agent conditions, but they do not by themselves establish pharmacologic synergy; combination-index analysis is presented separately in Section 2.6 [42].

At matched dose combinations, CP-PTX-KRAS G12D siRNA nanoparticles produced cytotoxicity that was similar to or modestly greater than the corresponding admixed PTX + Lipo-siRNA treatment. The nanoparticle and free-combination groups were not significantly different at 50 nM PTX + 10 nM siRNA, 100 nM PTX + 50 nM siRNA, or 100 nM PTX + 100 nM siRNA after Holm–Šídák adjustment. At 200 nM PTX + 100 nM siRNA, the nanoparticle group showed 11% ± 8.1% viability compared with 16% ± 7.1% for the free combination, with an adjusted *p* value < 0.01 (Figure 5). Thus, the selected-dose experiment supports comparable or modestly greater cytotoxicity with nanoparticle co-delivery, with a statistically significant difference between the two combination formats only at the highest matched dose shown.

Collectively, these data show that PTX/KRAS G12D-directed siRNA combinations were associated with greater cytotoxicity than the tested single-agent conditions, and that nanoparticle co-delivery was comparable to or modestly more cytotoxic than the admixed combination at selected doses. However, because no non-targeting siRNA control was included and KRAS mRNA or protein knockdown was not measured, the enhanced cytotoxicity cannot be attributed specifically to sequence-dependent KRAS G12D silencing. The present results therefore support a formulation-associated combination effect rather than a demonstrated KRAS-dependent mechanism. Direct target-engagement measurements, downstream signaling analyses, and appropriate non-targeting siRNA controls are required to establish mechanism.

### 2.6. Ratio-Dependent Cytotoxic Effects of CP-PTX-KRAS G12D siRNA NPs in PANC-1 Cells

To systematically evaluate the therapeutic interactions between paclitaxel (PTX) and KRAS G12D-targeting siRNA, we assessed cytotoxicity in PANC-1 cells following treatment with single agents, free-drug combinations, and CP-PTX-KRAS siRNA nanoparticles at defined PTX:siRNA molar ratios. Cell viability was measured after 72 h and normalized to untreated controls.

**Figure 6:**
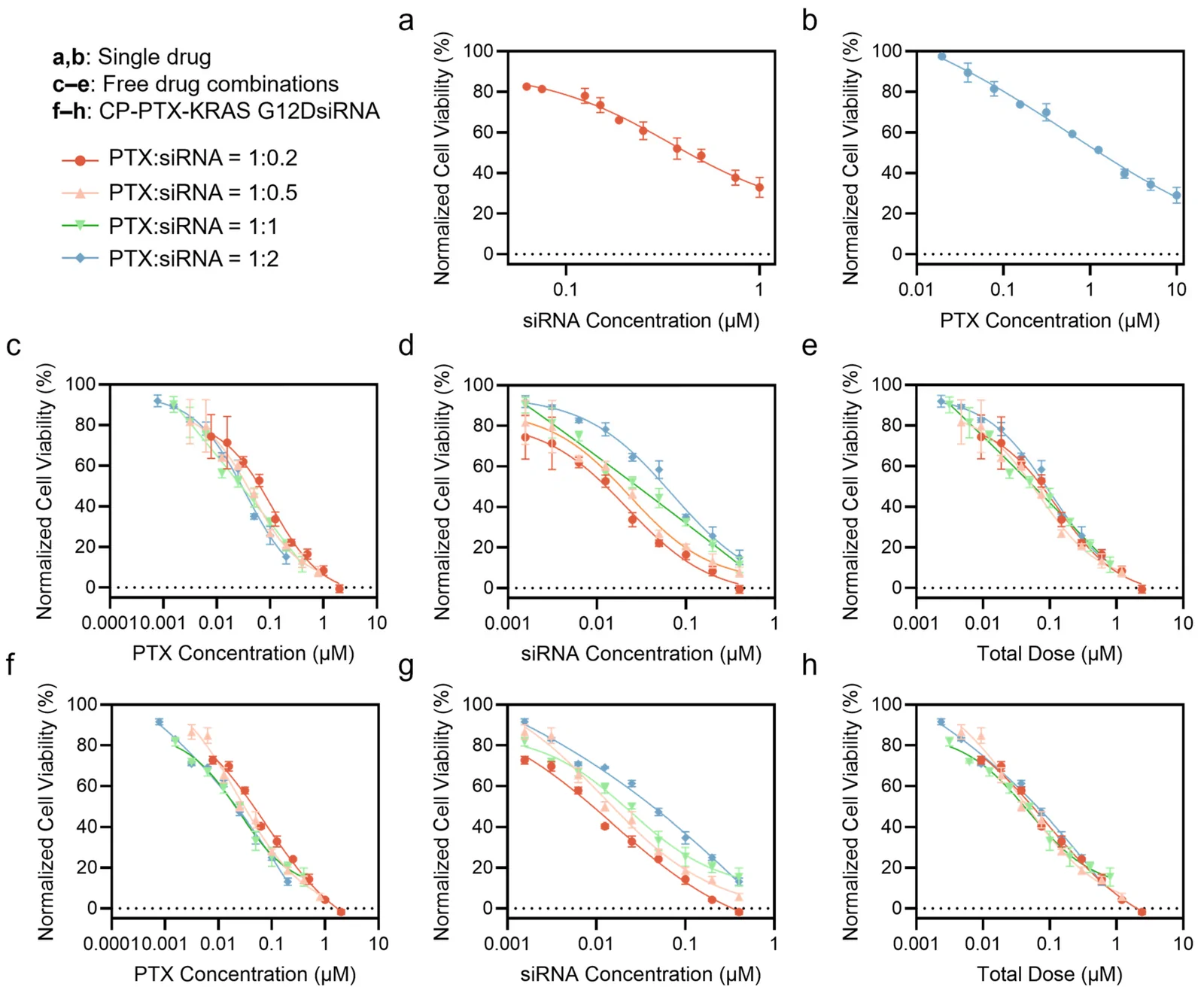
Dose–response analysis of PTX, KRAS-targeting siRNA, and CP-PTX-siRNA nanoparticles in PANC-1 cells. (**a**,**b**) Dose–response curves of single-agent treatments: (**a**) KRAS G12D siRNA and (**b**) free PTX, showing moderate, concentration-dependent cytotoxicity. (**c**–**e**) Dose–response curves of free PTX + KRAS siRNA combinations at fixed molar ratios (1:0.2, 1:0.5, 1:1, and 1:2), plotted as a function of (**c**) PTX concentration, (**d**) siRNA concentration, and (**e**) total dose. (**f**–**h**) Dose–response curves of CP-PTX-KRAS siRNA nanoparticles at corresponding PTX:siRNA molar ratios, plotted against (**f**) PTX concentration, (**g**) siRNA concentration, and (**h**) total dose. PANC-1 cells were treated for 72 h, and cell viability was measured using the Alamar Blue assay. Data represent mean ± standard deviation (SD) from three technical replicate wells within a single experiment and were normalized to untreated controls. Dose–response curves were fitted using a four-parameter logistic regression model in GraphPad Prism (Version 10.6.1).

Dose–response analysis of the individual agents demonstrated that both PTX and KRAS-targeting siRNA induced concentration-dependent reductions in cell viability, although the overall cytotoxic effects were limited when administered as monotherapies (Figure 6a,b). PTX exhibited moderate potency, while siRNA alone showed relatively weak cytotoxicity, consistent with its mechanism of targeting oncogenic signaling rather than directly inducing cell death. These results indicate that neither modality alone is sufficient to achieve strong therapeutic efficacy in this model.

In contrast, combining PTX and KRAS-targeting siRNA enhanced cytotoxic effects. Free-drug co-treatment resulted in steeper dose–response curves and lower cell viability compared to either agent alone (Figure 6c–e), indicating additive to synergistic interactions. This enhancement may be associated with the complementary activities of PTX-mediated cytotoxic stress and KRAS-targeting siRNA treatment.

To further improve co-delivery efficiency and therapeutic coordination, CP-PTX-KRAS siRNA nanoparticles were evaluated at fixed PTX:siRNA molar ratios (1:0.2, 1:0.5, 1:1, and 1:2, equivalent to CP-PTX:siRNA mass ratios of 1:0.1 to 1:1, respectively). As shown in Figure 6f–h, all nanoparticle formulations exhibited well-defined sigmoidal dose–response relationships when plotted against PTX concentration, siRNA concentration, or total dose, with strong curve fitting (R^2^ > 0.94), indicating consistent pharmacological behavior across formulations. The IC50 and CI analyses were derived from three technical replicate wells within a single experiment; therefore, they characterize calculated in vitro synergy for this experiment but do not establish biological reproducibility.

Quantitative analysis further demonstrated that nanoparticle-mediated co-delivery improves therapeutic potency compared to free-drug combinations. As summarized in Table 1, CP-PTX-KRAS siRNA nanoparticles consistently achieved lower total IC_50_ values than corresponding free-drug formulations across all tested ratios. For example, at the 1:0.2 ratio, the nanoparticle formulation exhibited a total IC_50_ of 0.0921 µM, compared to 0.133 µM for the free-drug combination, indicating a reduction in the effective dose required to achieve equivalent cytotoxicity. Similar trends were observed for the 1:0.5, 1:1, and 1:2 ratios, confirming improved efficiency of nanoparticle-mediated delivery.

To further characterize drug interactions, combination index (CI) analysis was performed using the Chou–Talalay method. As shown in Table 1, all nanoparticle formulations exhibited CI values below 1 (ranging from 0.317 to 0.477), indicating synergistic interactions between PTX and KRAS-targeting siRNA under the tested in vitro conditions. These values were consistently lower than or comparable to those observed for free-drug combinations, suggesting that co-delivery within a single nanoparticle may improve coordination of PTX and siRNA activity.

Importantly, both cytotoxic potency and calculated synergy were dependent on the PTX:siRNA ratio. The 1:0.2 formulation exhibited the most favorable profile, with the lowest CI value (0.317) and high potency, suggesting that a relatively small amount of siRNA may be sufficient to enhance PTX activity when efficiently co-delivered. Increasing the siRNA proportion beyond this ratio did not further improve efficacy and in some cases slightly reduced potency, highlighting the importance of ratio optimization rather than simply increasing total dose.

Overall, the dose–response and CI analyses provide a within-experiment comparison of PTX/KRAS G12D-directed siRNA formulations and identify ratio-dependent differences in fitted potency and calculated interaction. Independent biological replicates, direct KRAS target-engagement measurements, and non-targeting siRNA controls are required before concluding that the observed interaction is biologically reproducible or KRAS-specific.

### 2.7. Design Rationale, Mechanistic Insights, and Study Limitations of CP-PTX-siRNA Nanoparticles

The design of the CP-PTX-siRNA platform was driven by the challenge of co-delivering chemotherapeutic agents and nucleic acids, which possess distinct physicochemical properties and delivery requirements. Paclitaxel is hydrophobic and benefits from stable incorporation to reduce premature loss, whereas siRNA is hydrophilic, negatively charged, and susceptible to degradation. In contrast to systems that rely primarily on physical drug encapsulation, the CP-PTX design employs covalent conjugation of PTX to a chitosan–PEG backbone while preserving cationic functionality for electrostatic siRNA complexation. This modular architecture enables formulation optimization by adjusting CP-PTX:siRNA ratios and supports the formation of compact nanoparticles with favorable physicochemical properties. The TEM morphology, hydrodynamic size, zeta potential, and short-term physical stability in serum-containing medium together support the formation of nanoscale CP-PTX-siRNA complexes suitable for in vitro delivery studies. Although the current formulation generated compact nanoscale particles with favorable short-term stability, more uniform particle-size distributions are generally desirable for nanoparticle formulations to improve reproducibility, dosing consistency, and translational development. Therefore, further control of particle-size distribution will be considered in future formulation and dosage-form optimization studies.

With these formulation attributes, CP-PTX-siRNA nanoparticles generated intracellular Cy5-associated signal in Pan02 cells, while CP-Luc siRNA without PTX reduced luciferase luminescence in Pan02-Luc cells. In PANC-1 cells, PTX/KRAS G12D-directed siRNA formulations showed enhanced cytotoxicity relative to the tested single-agent conditions, and CI values below 1 were calculated within a single experiment. Several limitations constrain mechanistic interpretation: (i) KRAS mRNA/protein knockdown and downstream signaling were not directly measured; (ii) no non-targeting siRNA control was included; (iii) uptake and luciferase studies were performed in Pan02/Pan02-Luc rather than PANC-1 cells, so comparable uptake in PANC-1 is an untested assumption; (iv) the single-timepoint LysoTracker images cannot demonstrate or quantify endosomal escape; (v) the CP-PTX-Luc siRNA luminescence readout is confounded by potential PTX effects on cell number or metabolism; and (vi) the dose–response and CI analyses used technical triplicates from one independent experiment and therefore do not establish biological reproducibility. PTX release kinetics from the current CP-PTX-siRNA formulation and direct siRNA complexation/loading efficiency also remain unmeasured. Future studies should address these limitations through independent biological replication, direct KRAS target-engagement and downstream-pathway assays, non-targeting siRNA controls, uptake studies in PANC-1 cells, quantitative intracellular-trafficking analysis, PTX release measurements, siRNA protection/loading assays, and in vivo evaluation.

Accordingly, the present findings support CP-PTX-siRNA nanoparticles as a formulation platform for PTX/siRNA co-delivery and provide a basis for independent mechanistic and in vivo validation.

## 3. Materials and Methods

### 3.1. Materials

All chemicals were purchased from Sigma-Aldrich (St. Louis, MO, USA) unless otherwise stated. Chitosan powders were purchased from ChitoLytic Biotech Ltd. (Yancheng, China) (MW ~3900, deacetylation rate 91.5%). PTX was purchased from LC Laboratories (Woburn, MA, USA). Bovine serum albumin, wheat germ agglutinin–Alexa Fluor 555 conjugate, 8-well Nunc™ Lab-Tek™ II Chambered Coverglass and eBioscience™ Calcein AM Viability Dye (UltraPure Grade) were purchased from ThermoFisher Scientific (Waltham, MA, USA). NucBlue DAPI reagent, Dulbecco’s Modified Eagle Medium (DMEM), and RPMI 1640 cell culture medium were purchased from Invitrogen (Carlsbad, CA, USA). HyClone characterized fetal bovine serum (FBS) was purchased from GE Healthcare Life Sciences (Pittsburgh, PA, USA). SpectraPOR7 1 kDa RC dialysis tubing was purchased from Repligen Corp (Waltham, MA, USA).

Pan02 and Pan02-Luciferase (Pan02-Luc) murine pancreatic cancer cells were obtained from the National Cancer Institute (NCI) Division of Cancer Treatment and Diagnosis Tumor Repository (Frederick, MD, USA). Human PANC-1 pancreatic cancer cells were purchased from the American Type Culture Collection (ATCC, Manassas, VA, USA). Luciferase-targeting siRNA (sense: 5′-CUUACGCUGAGUACUUCGAdTdT-3′) and KRAS G12D-targeting siRNA (sense: 5′-GUUGGAGCUGAUGGCGUAGdTdT-3′) were purchased from Dharmacon, Inc. (Lafayette, CO, USA).

### 3.2. Synthesis of Paclitaxel-Carboxylic Acid (PTX-COOH)

PTX-COOH was prepared by modifying a previously established method [43]. Briefly, PTX (50 mg) was mixed with succinic anhydride (11.8 mg) in 6 mL of chloroform containing 56.9 µL of pyridine. The solution was stirred at ambient temperature for 24 h to allow complete derivatization. After the reaction, the solvent was removed by vacuum drying overnight. The resulting solid was collected and washed three times with deionized water using centrifugation (4000× *g*, 2 min) to remove unreacted reagents. The washed residue was redissolved in 2 mL of acetone and transferred to a 15 mL conical tube. Water was slowly added (~10 mL total) until the solution became visibly turbid, at which point the mixture was centrifuged at 1000 ×g for 10 min. The pellet was resuspended in 2 mL of deionized water and freeze-dried to yield purified PTX-COOH.

### 3.3. Conjugation of PTX-COOH to Chitosan–PEG

Chitosan–PEG (CP) was synthesized as described previously [39]. Briefly, aldehyde-functionalized PEG (2000 MW) was conjugated to chitosan through reductive amination between the aldehyde group of PEG and primary amine groups on the chitosan backbone in the presence of sodium cyanoborohydride. This reaction forms a stable secondary amine linkage between PEG and chitosan, generating the CP polymer used for subsequent PTX conjugation. For PTX conjugation, PTX-COOH (4.3 mg) was activated by reacting with EDC (3.2 mg) and NHS (1.6 mg) in 0.5 mL of DMSO for 3 h on a rocker. Separately, CP (20 mg) was dissolved in 0.8 mL of 0.1 M sodium bicarbonate buffer (pH 8.5), then diluted with 1.6 mL of DMSO. The activated PTX-COOH solution was gradually added to the CP solution under stirring and allowed to react overnight.

To reduce the DMSO concentration, 8 mL of deionized water was added dropwise to the mixture. The solution was then transferred into dialysis tubing (1 kDa MWCO, RC) and dialyzed against 2 L of deionized water for 24 h with changes at 1, 3, and 7 h. After dialysis, the full content—including any precipitates—was subjected to bath sonication using a Fisher Sonic Dismembrator (Model 500) (Waltham, MA, USA) for 10 min at 40% amplitude (pulse mode: 10 s on, 5 s off). The mixture was centrifuged at 20,000× *g* for 10 min, and the supernatant containing CP-PTX was collected and stored at 4 °C.

To fluorescently label the nanoparticles, 2.7 mL of CP-PTX solution was mixed with 0.3 mL of 10× PBS (pH 7.4), and 10 µL of NHS-Cy5 (5 mg/mL in DMSO) was added. The reaction proceeded for 2 h at room temperature with rocking. Excess dye was removed by 24 h dialysis against deionized water using 1 kDa MWCO tubing, with three water changes. The concentration of CP-PTX and Cy5-labeled CP-PTX was determined by freeze-drying 10 mL of solution and weighing the dry mass.

### 3.4. Nanoparticle Co-Loading with siRNA

For siRNA loading, siRNA stock solution (20 μM in RNase-free water, luciferase or KRAS G12D-targeted siRNA) was mixed with CP-PTX nanoparticles at predetermined ratios to form siRNA-loaded nanocomplexes. All components were mixed in nuclease-free water. Complexation was facilitated by water bath sonication at 60 kHz, using a pulsed cycle of 10 s on and 5 s off for a total of 5 min at 25 °C. The CP-PTX-siRNA nanoparticles were prepared immediately before use, and the resulting formulations were used without an additional isolation or purification step.

### 3.5. ^1^H Nuclear Magnetic Resonance (1H NMR) Characterization

NMR spectra were acquired using a Bruker AVANCE III 500 MHz spectrometer operating at a ^1^H frequency of 499.65 MHz. CP and CP-PTX samples were dissolved in D_2_O, while PTX was measured in DMSO-d_6_. Acquisition parameters included: 96 scans (NS), acquisition time (AQ) of 2.34 s, and time domain (TD) of 32,678 points.

To quantify PTX content in the conjugate, a dried sample was redissolved in 0.9 mL of D_2_O and mixed with 0.1 mL of TSP (3-(Trimethylsilyl) propionic-2,2,3,3-d_4_ acid sodium salt) solution at 17.2 mg/mL. Peak integrations at δ = 7.75 ppm (PTX, 2H) and δ = 0.00 ppm (TSP, 9H) were used to calculate PTX content via the equation [31]:

$$m_{D\;=\;}\frac{m_{IS}P_{IS}I_{D}M_{D}}{M_{IS}P_{D}I_{IS}}$$

where

m_D_: mass of PTX

m_IS_: mass of internal standard (TSP)

P_D_, PIS: number of protons corresponding to the drug and internal standard peaks

I_D_, IIS: integrated peak areas

M_D_, M_IS_: molecular weights of PTX and TSP, respectively

### 3.6. Dynamic Light Scattering

Hydrodynamic diameter and zeta potential were characterized using a Zetasizer Nano-ZS (Malvern Instruments, MalvernWorcestershire, UK). Samples were prepared in deionized water or 20 mM HEPES buffer (pH 6.8) as indicated. Measurements were performed at room temperature using disposable cuvettes for size analysis and folded capillary cells for zeta potential measurements. Data were analyzed using the instrument software to obtain intensity-weighted hydrodynamic size distributions and zeta potential values.

For the 5-day stability study, optimized CP-PTX-siRNA nanoparticles at a CP-PTX:siRNA mass ratio of 1:0.1 were incubated in RPMI cell culture medium supplemented with 10% FBS at 37 °C. Hydrodynamic size was measured daily by DLS for 5 days. Because serum-containing medium contains proteins and other scattering components, the values obtained under these conditions are reported as apparent hydrodynamic diameters.

### 3.7. Transmission Electron Microscopy (TEM)

Nanoparticle morphology of the optimized CP-PTX-siRNA formulation at a CP-PTX:siRNA mass ratio of 1:0.1 was examined via TEM imaging. TEM samples were prepared by the addition of 4 μL of CP-PTX-siRNA solution to a Formvar/carbon coated 300-mesh copper grid (Ted Pella, Inc., Redding, CA, USA) and allowed to air dry. TEM images were acquired on a Tecnai G2 F20 electron microscope (FEI, Hillsboro, OR, USA) operating at a voltage of 200 kV.

### 3.8. Cell Culture

Pan02 and Pan02-Luc cells were maintained in RPMI medium supplemented with 10% fetal bovine serum (FBS) and 1% penicillin–streptomycin. PANC-1 cells were cultured in DMEM with 10% FBS and 1× penicillin–streptomycin. All cell lines were maintained in a humidified incubator at 37 °C with 5% CO_2_ and were routinely passaged at 70–80% confluency using standard trypsinization procedures. Cells were regularly monitored for morphology and viability and were used within a limited number of passages to ensure experimental consistency.

### 3.9. Cell Uptake and LysoTracker Colocalization of CP-PTX-siRNA Nanoparticles

For the CP-PTX-siRNA nanoparticle uptake study, Pan02 cells were seeded at 8000 cells per well in an 8-well chambered coverglass and incubated for 24 h. Cells were then treated with 100 µg/mL Cy5-labeled CP-PTX-Luc siRNA nanoparticles for 2 h at 37 °C. After treatment, cells were fixed with 4% paraformaldehyde, cell membranes were stained with wheat germ agglutinin–Alexa Fluor 555, and nuclei were counterstained with DAPI. Cellular association/internalization and intracellular distribution were assessed qualitatively by confocal laser scanning microscopy.

For the LysoTracker colocalization study, Pan02 cells were seeded at 8000 cells per well in an 8-well chambered coverglass and incubated for 24 h. Cells were treated with 100 µg/mL Cy5-labeled CP-PTX-Luc siRNA nanoparticles for 4 h at 37 °C. LysoTracker Red DND reagent (450 nM) was added 1 h before fixation. Cells were washed three times with cold PBS and fixed with 4% paraformaldehyde in PBS for 15 min at room temperature, followed by three additional PBS washes. NucBlue FixCell ReadyProbe DAPI reagent was diluted 10-fold in cold PBS and 100 µL was added to each well. Confocal images were acquired using a Leica SP8X confocal laser scanning microscope (Wetzlar, Germany). This experiment was used for qualitative assessment of spatial overlap with LysoTracker-positive compartments; no quantitative endosomal-escape measurement was performed.

### 3.10. Luciferase Readout Following CP-Based siRNA Delivery

Pan02-Luc cells were seeded at 15,000 cells per well in 96-well plates and incubated for 24 h. Cells were then treated for 24 h with Lipo alone, Lipo-Luc siRNA, CP alone, CP-Luc siRNA, or CP-PTX-Luc siRNA, matching the groups shown in Figure 4c. Luc siRNA was used at 200 nM in all siRNA-containing groups, and carrier-only controls were applied at the amounts used in the corresponding siRNA formulations. Lipofectamine RNAiMAX (Thermo Fisher Scientific) was used for the Lipo-Luc siRNA positive-control formulation according to the manufacturer’s protocol. Luciferase activity was assessed using a SpectraMax i3 microplate reader (Molecular Devices, Sunnyvale, CA, USA) and a Xenogen IVIS 200 imaging system (PerkinElmer, Waltham, MA, USA). D-luciferin was added to each well at a final concentration of 3 mg/mL. For plate-reader measurements, luminescence was recorded after a 5 min incubation. For IVIS imaging, images were acquired immediately after luciferin addition using a 1 min exposure, medium binning, f/stop 1, open emission filter, and field of view C; subject height was set to 0.5 cm. Total photon flux was quantified using Living Image software (version 3.0) and normalized to untreated controls. No parallel normalization to cell viability, cell number, or total protein was performed; accordingly, the PTX-containing group was not used as independent evidence of siRNA-mediated knockdown.

### 3.11. Cytotoxicity of PTX/KRAS G12D-Directed siRNA Formulations

PANC-1 cells were seeded at 10,000 cells per well in a 96-well plate and allowed to attach for 24 h. Cells were then treated with PTX (dissolved in DMSO and diluted in media) or siRNA (delivered using Lipofectamine) as single agents, an admixed free-drug combination of PTX and siRNA, or CP-PTX–KRAS G12D siRNA NPs at various ratios and concentrations. Cells were incubated for 72 h at 37 °C. After treatment, Alamar Blue reagent was added to each well, and fluorescence was measured using a SpectraMax i3 microplate reader (Molecular Devices, Sunnyvale, CA, USA) with 550 nm excitation and 590 nm emission to quantify cell viability. Each treatment condition was analyzed using three technical replicate wells within a single experiment, and the data are presented as mean ± SD of these technical replicates.

Combination index analysis was performed using the Chou–Talalay method. Cell viability data from PTX alone, KRAS-targeting siRNA alone, free-drug combinations, and CP-PTX-KRAS siRNA nanoparticle treatments were used to determine dose–response relationships and IC50-equivalent concentrations. For each combination, the corresponding PTX and siRNA concentrations producing an equivalent inhibitory effect were used to calculate CI values. CI values were interpreted as follows: CI < 1 indicates synergistic interactions, CI = 1 indicates an additive effect, and CI > 1 indicates antagonistic interactions. The dose–response curves and CI values were derived from three technical replicate wells within a single independent experiment; no biological-replicate estimate of variability in IC50 or CI was available.

### 3.12. Statistical Analysis

All data are presented as mean ± standard deviation (SD), unless otherwise indicated. Statistical significance was assessed using one-way ANOVA followed by appropriate post hoc tests for multiple comparisons. For pairwise comparisons, unpaired two-tailed Student’s *t*-tests were used. A *p*-value less than 0.05 was considered statistically significant. Significance levels are indicated as follows: *p* < 0.05 (*), *p* < 0.01 (**), *p* < 0.001 (***), and *p* < 0.0001 (****). For the matched-dose pairwise comparisons shown in Figure 5, *p* values were adjusted using the Holm–Šídák method; these within-experiment comparisons are exploratory.

## 4. Conclusions

This study developed a chitosan–polyethylene-glycol-based nanoparticle platform for co-delivery of paclitaxel and small interfering RNA. Covalent PTX conjugation to the CP backbone and subsequent electrostatic siRNA association generated a tunable two-cargo formulation with nanoscale dimensions and short-term colloidal stability under the conditions tested.

Confocal imaging showed intracellular Cy5-associated CP-PTX-siRNA signal in Pan02 cells. In Pan02-Luc cells, CP-Luc siRNA reduced luciferase luminescence, supporting the capacity of the CP carrier to deliver biologically active siRNA. In contrast, the lower luminescence observed with the PTX-containing CP-PTX-Luc siRNA formulation cannot be interpreted as independent evidence of RNA interference because the readout was not normalized to cell viability, cell number, or total protein. Likewise, the single 4 h LysoTracker images show possible partial spatial separation but do not establish or quantify endosomal escape.

In KRAS G12D-mutant PANC-1 cells, PTX/KRAS G12D-directed siRNA combinations were more cytotoxic than the tested single-agent conditions, and nanoparticle formulations yielded CI values below 1 across several PTX:siRNA ratios, yet a direct cause of the enhanced cytotoxicity by KRAS silencing requires further evidence of KRAS knockdown and a non-targeting siRNA control. In addition, the IC50 and CI analyses were based on technical triplicates from one independent experiment and therefore do not demonstrate biological reproducibility. Uptake of the formulation was also not directly measured in PANC-1 cells.

Overall, the data support the feasibility of the CP platform for coordinated PTX/siRNA delivery and identify formulation ratios for further study. Independent biological replication, direct KRAS target-engagement assays, non-targeting siRNA controls, PANC-1 uptake studies, quantitative intracellular-trafficking measurements, and in vivo evaluation will be required to establish mechanism, reproducibility, and translational relevance.

## Figures and Tables

**Table 1:** Combination Index (CI) values of PTX and siRNA administered either as admix drug co-treatment or as CP-PTX-siRNA NPs at varying PTX:siRNA molar ratios (1:0.2, 1:0.5, 1:1, and 1:2). For each combination, the single-agent IC_50_ values used for the Chou–Talalay combination index analysis based on the Chou–Talalay method were obtained from the dose–response curves shown in Figure 6 [42]. All fitted values and CI calculations are based on three technical replicate wells from a single independent experiment; CI < 1 denotes calculated synergy within this experiment but does not establish biological reproducibility.

PTX:siRNA Ratio	Formulation	PTX IC_50_ (µM)	siRNA IC_50_ (µM)	Total Dose IC_50_ (µM)	CI Value	Interpretation
PTX only	Free drug	0.547	-	-	-	-
siRNA only	Free drug	-	0.278	-	-	-
1:0.2	Free drug	0.111	0.0218	0.133	0.400	Synergistic
	NPs	0.0783	0.0139	0.0921	0.317	Synergistic
1:0.5	Free drug	0.0469	0.0230	0.0700	0.506	Synergistic
	NPs	0.0233	0.0111	0.0344	0.386	Synergistic
1:1	Free drug	0.0363	0.0617	0.0980	0.668	Synergistic
	NPs	0.0241	0.0184	0.0425	0.449	Synergistic
1:2	Free drug	0.0354	0.0720	0.107	0.684	Synergistic
	NPs	0.0467	0.0184	0.0651	0.477	Synergistic

## Data Availability

The original contributions presented in this study are included in the article. Further inquiries can be directed to the corresponding authors.
